# Editorial: Genetic and Gene Regulation Underlying Sex Differences in Cardiovascular Disease

**DOI:** 10.3389/fcvm.2022.881154

**Published:** 2022-04-11

**Authors:** Emma L. Robinson, Susana Novella

**Affiliations:** ^1^Division of Cardiology and Consortium for Fibrosis Research & Translation, Department of Medicine, University of Colorado Anschutz Medical Campus, Aurora, CO, United States; ^2^Department of Physiology, INCLIVA Biomedical Research Institute, University of Valencia, Valencia, Spain

**Keywords:** sex, sex hormones, epigenetics, estrogen, androgen

Epidemiology and clinical studies demonstrate clear sex differences for cardiovascular risk, prevalence, prognosis, pathophysiological manifestation, and response to treatment in cardiovascular diseases (CVD). These differences in cardiovascular pathophysiology, driven by core transcriptional, biochemical and structural changes in the heart and vasculature, involve both hormonal status and gene regulation. This Research Topic presents a collection of original research and reviews that address different aspects of cardiovascular implications of sex-related differences.

Traditionally, sex bias in CVD has been attributed to differential genetic and epigenetic factors under the hormonal influence. However, the cardiac genome is already functionally distinct between males and females, with genes linked to the X and Y chromosomes encoding regulatory factors expressed at early developmental stage. Deegan et al. focus precisely on these sex chromosome-linked genes and their targets, but also on the important differences in transcriptomic profile between the sexes. Beginning with the influence of sex hormones and their regulatory pathways on healthy hearts and heart disease in males and females, they also emphasize the importance of these cardiac sex differences from early development stages, when sex chromosomes influence the expression and epigenetic patterns by several mechanisms, as another point to consider in the study of regulation of sex-dependent cardiovascular mechanisms. In this regard, inherited hypertrophic cardiomyopathies due to pathogenic mutations in sarcomeric proteins exhibit skewed incidence, indicating that sex-specific factors can compensate for the genetic defects and contribute to the penetrance of the disease. Females present with more severe diastolic dysfunction, and cellular differences underlying sex-differences in the clinical presentation of this pathology are not well-known either. Thorough a proteomic analysis performed in cardiac tissue from males and females harboring a sarcomere mutation, Schuldt et al. showed higher levels of tubulin and heat shock proteins in females. The elevated proteins levels of tubulin correlated with more severe diastolic dysfunction in females. The gene Duchenne muscular dystrophy (*DMD*) is also more highly expressed in females than in males. This gene encodes for dystrophin and is located on the X-chromosome. Mutations in *DMD* have been associated with muscular dystrophies and X-linked dilated cardiomyopathy (1), which again highlights the need to study the chromosomal contribution to cardiovascular sex differences.

Sex-differences are also observed in the incidence of postoperative delirum (POD) after a cardiac surgery. In a retrospective case-control study performed by Wang et al., male gender is associated with an increased risk of POD following cardiac valve surgery, and along with gender, the incidence of POD was also higher in males under the age of 60 years. Thus, both age and sex should be considered in POD preventive measures in patients undergoing cardiac valve surgery.

Sex hormone studies have been outstanding in determining sex differences at the cardiovascular level. Among them, both estradiol and testosterone target the endothelium and modulate endothelial function through genomic and non-genomic actions. Testosterone has been also related to beneficial effects in the development of CVD, as higher plasma testosterone is protective, and the decline in the testosterone concentrations with aging increase cardiovascular events (2). To unravel the underlying mechanisms linking low endogenous testosterone in males with atherosclerosis, Groepenhoff et al. performed a study of plasma testosterone levels in males with advanced atherosclerotic disease in relation to gene expression in atherosclerotic plaque tissue in the frame of Athero-Express Sdudy (3). Despite the high relative expression of the androgen receptor in this tissue, neither gene expression profile nor regulatory network in late-stage atherosclerotic plaques were related with plasma testosterone levels. Thus, more studies in the effect of testosterone on atherosclerosis are need to explain the clinical outcomes observed in these patients.

Male sex hormones have been also studied in prostate cancer patients undergoing androgen deprivation therapy, which favors a pro-inflammatory and pro-oxidant environment and an increased risk of cardiovascular disease. There remains an unmet need for basic research investigations to elucidate the influence of sex hormones on the cardiovascular system, such as that presented by Álvarez-Maestro et al., in which authors stablished a relationship between the androgen deprivation therapy in prostate cancer patients and circulating levels of thromboxane A_2_ (TXA_2_). The incremental levels of TXA_2_ in these patients with androgen deprivation could impact negatively at cardiovascular system increasing the risk, as TXA_2_ also induced vascular dysfunction through increased HO-1, COX-2, and p-ERK1/2 expression in aortic segments of healthy male rats, supporting a pro-inflammatory environment induced by TXA_2_.

Mostly probably, the majority of studies using human data and samples that have been carried out to determine the effect of sex differences on the cardiovascular system have been from specimen from post-menopausal females. The increase in cardiovascular diseases during menopause, and the existing controversy regarding the adequacy of hormone replacement therapy in the prevention of CVD has sparked interest in food science and nutraceutical biochemistry. In this sense, Wickham et al. present an interesting research work evaluating the impact of supplementation with fermented red clover extract on the vascular health. Red clover is rich in phytoestrogen and in isoflavones biochanin A and formonentin (4). Results from this study indicate that with just two weeks of supplementation with red clover extract reduced the vascular inflammation marker VCAM-1 expression and improves vascular inflammation in early post-menopausal females, opening a window to future investigations on red clover supplementation for an improvement of vascular and metabolic health in females.

Overall, the articles published in this Research Topic highlight the importance of consider sex in basic and clinical research, from chromosomes to sex hormones, and in gene expression and genetic regulation of the cardiovascular pathophysiology (Figure 1).

**Figure 1:**
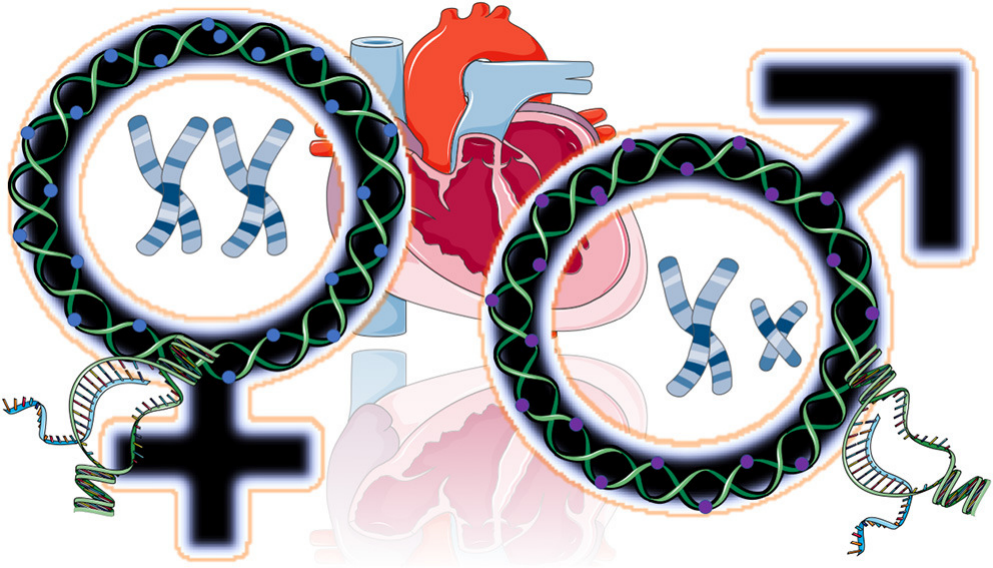
Illustrative figure of the sex differences in cardiovascular genetics.

## Author Contributions

ER and SN drafted and edited the editorial. Both authors contributed to the article and approved the submitted version.

## Funding

ER was supported by an American Heart Association Postdoctoral fellowship (#829504). SN was supported by Spanish Ministerio de Ciencia e Innovación, Instituto de Salud Carlos III—FEDER-ERDF (grants PI16/00229 and PI19/01714), and Generalitat Valenciana (AICO 2020/030).

## Conflict of Interest

The authors declare that the research was conducted in the absence of any commercial or financial relationships that could be construed as a potential conflict of interest.

## Publisher's Note

All claims expressed in this article are solely those of the authors and do not necessarily represent those of their affiliated organizations, or those of the publisher, the editors and the reviewers. Any product that may be evaluated in this article, or claim that may be made by its manufacturer, is not guaranteed or endorsed by the publisher.
